# Lip piercing-related prosthetic aortic graft infection and mediastinitis in filaminopathy: A case report

**DOI:** 10.1016/j.jvscit.2026.102226

**Published:** 2026-03-12

**Authors:** Solène Brouder, Corina Mirea, Camille Zamperini, Franck Zheng, Philippe Billaud, Anne Lejay, Dominique Stephan, Elena-Mihaela Cordeanu

**Affiliations:** a Department of Hypertension and Vascular Diseases, Clinical Pharmacology, Strasbourg University Hospitals, Strasbourg, France; b Department of Cardiac Surgery, Strasbourg University Hospitals, Strasbourg, France; c Department of Vascular Surgery and Kidney Transplantation, Strasbourg University Hospitals, Strasbourg, France

**Keywords:** Filaminopathy A, Mediastinitis, Prosthetic aortic graft infection, Lip piercing, Vascular prosthesis, Cryopreserved aortic allograft, Heritable connective tissue disorder, Antibiotic prophylaxis

## Abstract

We report a rare case in which a lip piercing led to *Staphylococcus aureus* bacteremia and subsequent prosthetic aortic graft infection complicated by anterior mediastinitis in a patient with filaminopathy A. A 32-year-old woman, who had undergone valve-sparing aortic root and arch replacement with a hybrid thoracic stent graft 5 years earlier, presented 3 weeks after a lip piercing with fever, chest pain, and elevated inflammatory markers. Computed tomography revealed a periprosthetic fluid collection and mediastinal inflammation consistent with graft infection. Blood cultures grew methicillin-sensitive *S aureus*. Despite early intravenous antibiotics, rapid progression to anterior mediastinitis and a large retrosternal abscess mandated radical surgical explantation of all prosthetic material and in situ reconstruction using multiple cryopreserved aortic allografts. She completed 6 weeks of intravenous cefazoline followed by prolonged oral antimicrobial therapy. At 4 months, imaging showed patent reconstruction and no residual infection. This case underscores the potential increased risk in patients with heritable aortopathies and vascular prostheses to bacteremia-related complications and highlights the importance of preventive counseling regarding procedures that may cause transient bacteremia, including oral piercings.

Filaminopathy A is a rare X-linked connective tissue disorder caused by *FLNA* mutations, resulting in cardiovascular manifestations including aortic aneurysms, valvular dysfunction, and increased vascular fragility.^1^, ^2^, ^3^ Patients with this condition often require early vascular interventions, including aortic root replacement, to prevent life-threatening complications. Prosthetic material confers lifelong susceptibility to hematogenous infection. Prosthetic aortic graft infections represent a devastating complication with mortality rates ranging from 10% to 75%, depending on the location and extent of infection.^4^ Although most graft infections result from perioperative contamination or hematogenous seeding from distant sites, oral procedures have been increasingly recognized as potential sources of bacteremia leading to prosthetic infections.^5^ Body piercings, particularly in the oral region, carry inherent risks of local and systemic infections.^6^^,^^7^ The oral cavity harbors diverse bacterial flora, and piercing procedures can introduce these micro-organisms into the bloodstream, potentially seeding distant prosthetic materials. Despite the growing popularity of body modifications, there is limited literature addressing the risks in patients with vascular prostheses undergoing such procedures. Although usually inconsequential, transient bacteremia represents a major threat in patients with prosthetic aortic material or heritable aortopathies, yet this risk is rarely addressed in routine clinical counselling. We describe a unique case of prosthetic aortic graft infection and mediastinitis secondary to lip piercing in a patient with filaminopathy A, highlighting the importance of patient education regarding infection prevention in this high-risk population.

## Case report

A 32-year-old woman with genetically confirmed filaminopathy A had a longstanding history of multiple systemic manifestations of the disorder. At age 16, she presented with seizures, and magnetic resonance imaging of the brain revealed periventricular nodular heterotopia, leading to the identification of a heterozygous pathogenic variant in the *FLNA* gene (c.62_64TCG exon 2, p.Val21dup). Over time, her vascular phenotype evolved to include thoracic aortic aneurysm, splenic and popliteal artery aneurysms, superior mesenteric artery ectasia, coronary artery abnormalities, and an aberrant retroesophageal right subclavian artery (arteria lusoria). The splenic and popliteal aneurysms and superior mesenteric artery ectasia were managed conservatively with close duplex and computed tomography angiography (CTA) surveillance. There was no history of infectious complications involving these vascular lesions. Additional systemic features comprised recurrent joint subluxations, moderate scoliosis, and centrilobular emphysema despite never having smoked. At age 27, she underwent valve-sparing aortic root replacement (David procedure) and total arch replacement using a hybrid Elephant Trunk technique with a 30- to 36-100 mm Thoraflex stented prosthesis, performed for severe thoracic aortic dilatation (sinus of Valsalva 75 mm) with associated severe aortic regurgitation.

**Fig 1:**
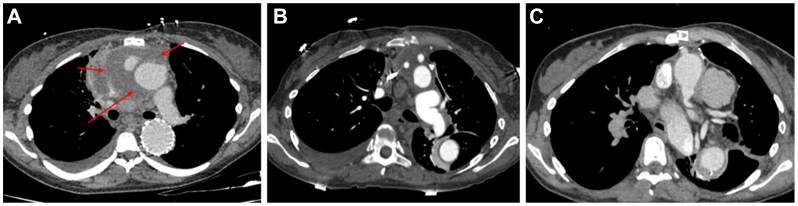
Computed tomography evolution of prosthetic aortic graft infection and postoperative reconstruction. Contrast-enhanced computed tomography angiography (CTA), axial views. **(A)** At admission, extensive mediastinal infiltration and circumferential periprosthetic fluid collection surrounding the ascending aortic graft, consistent with purulent mediastinitis (*red arrows*). **(B)** CTA 22 days after complete explantation of the infected Thoraflex and cTAG prostheses and in situ reconstruction with cryopreserved aortic allografts, showing patent reconstruction and resolution of mediastinal collections. **(C)** Four-month follow-up CTA demonstrating stable allograft reconstruction and absence of residual infection.

**Fig 2:**
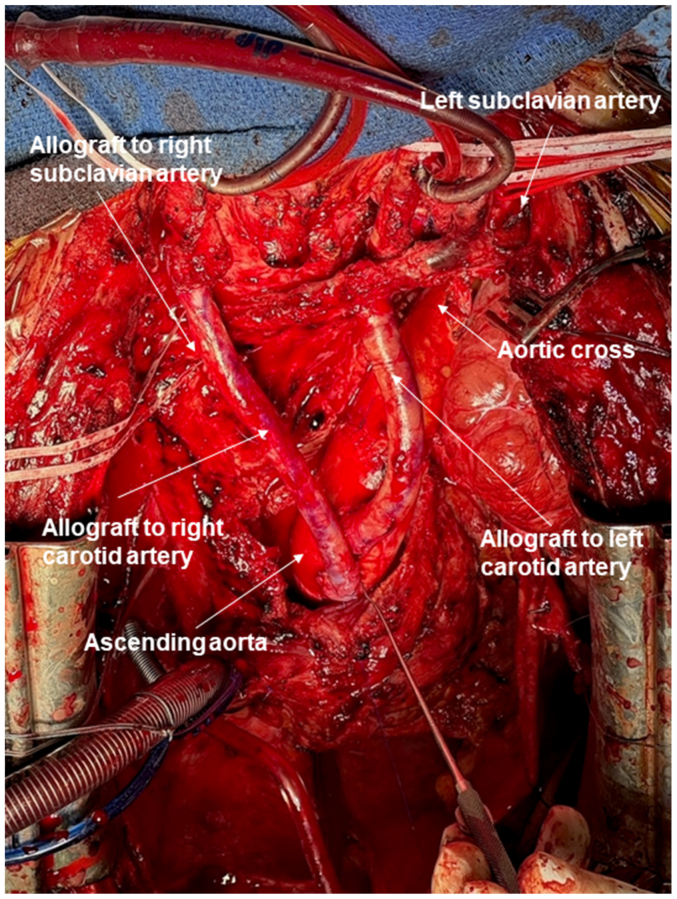
Intraoperative view after complete excision of the infected prosthetic graft and in situ reconstruction with cryopreserved aortic allografts. Operative photograph illustrating the reimplantation of the supra-aortic trunks performed in zone 0. The right-sided allograft directed posteriorly supplies the subclavian artery (allograft to right subclavian artery), while a second allograft is anastomosed to the right carotid artery (allograft to right carotid artery). The left-sided carotid reconstruction (allograft to left carotid artery) is indicated on the opposite side, but partially obscured in this operative view. This configuration was designed to facilitate potential future endovascular treatment in case of late allograft degeneration.

At age 32, she underwent a lip piercing without antibiotic prophylaxis. According to the patient's account and information obtained from the referring physicians, the piercing site became infected within the following days, with localized purulent inflammation and secondary oropharyngeal soft tissue involvement. The infected piercing was removed. This was not a deep-seated cervicofacial cellulitis or a discrete abscess requiring surgical drainage, and no dedicated cervical or oropharyngeal imaging was performed at that stage. Approximately 3 weeks after the lip piercing, on January 4, 2025, she was admitted to an external hospital in Germany with fever (39.5 °C) and right parasternal chest pain radiating to the back and neck. Blood cultures obtained during this admission grew methicillin-sensitive *Staphylococcus aureus* (MSSA), and intravenous antibiotic therapy was promptly initiated (ampicillin-sulbactam 3 g three times daily, subsequently switched to cefazolin 2 g four times daily and fosfomycin 5 g three times daily). Computed tomography demonstrated a new retrosternal fluid collection consistent with mediastinitis, and transesophageal echocardiography suggested aortic valve endocarditis. She was transferred to our center on January 10, 2025, for management of suspected prosthetic graft infection. On admission, CTA excluded aortic dissection but demonstrated a periaortic fluid collection surrounding the ascending aortic prosthesis (Fig 1). Repeat blood cultures confirmed persistent MSSA bacteremia. Transthoracic echocardiography showed leaflet thickening and a 15-mm periprosthetic collection. Despite high-dose intravenous cefazoline (8 g/d), serial CTA demonstrated rapid progression of a 2-cm retrosternal abscess—45 × 32 × 78 mm with mediastinal extension and superior vena cava compression—confirming prosthetic graft infection with anterior mediastinitis. After emergency coordination with tissue banks in Brussels and Besançon, radical surgery was performed on January 23, 2025 (day 19 after initial admission, day 13 after transfer). Intraoperative findings included approximately 150 mL of purulent fluid, complete infection of both the Thoraflex prosthesis and the cTAG endograft with mature biofilm, and multiple mediastinal abscesses. Under cardiopulmonary bypass with moderate hypothermia, all infected material was excised, including the native aortic valve. Reconstruction was performed using cryopreserved aortic allografts: a Yacoub root replacement, a 28-mm aortic arch allograft, and separate allografts for reimplantation of each supra-aortic vessel in zone 0 to allow future thoracic endovascular repair should aneurysmal degeneration of the allografts occur. Intraoperatively, the brachiocephalic trunks were reconstructed individually on each side. The operative view (Fig 2) illustrates the right-sided allograft coursing posteriorly toward the subclavian artery and anastomosed to the right carotid artery; left-sided reconstruction was performed in a similar manner. A perimembranous ventricular septal defect was closed with a bovine pericardial patch. The postoperative course was notable for cardiogenic shock requiring noradrenaline and dobutamine, left recurrent laryngeal nerve palsy, left diaphragmatic paralysis, paroxysmal atrial fibrillation on postoperative day 3 treated with amiodarone, and a superficial sternal wound infection on postoperative day 12. CTA identified a 29 × 17 mm pseudoaneurysm at the posterior aspect of the ascending aorta, which decreased to 20 mm and later stabilized at 24 mm. Extensive left iliofemoral deep vein thrombosis extending to the inferior vena cava occurred after cannulation and extracorporeal circulation and was treated with tinzaparin and later transitioned to apixaban. Antibiotic therapy consisted of 6 weeks of intravenous cefazoline followed by oral trimethoprim-sulfamethoxazole combined with rifampicin 600 mg/d. One month postoperatively, she had achieved clinical stabilization, with negative blood cultures and normalization of inflammatory markers. At the 4-month follow-up (April 2025), CTA confirmed patent allograft reconstruction with stable dimensions and regression of the pseudoaneurysm to 16 × 15 mm. Neurological deficits related to recurrent laryngeal and phrenic nerve injury persisted, but were undergoing active rehabilitation. A duplex ultrasound examination of the supra-aortic trunks performed 9 months after surgery demonstrated anastomotic stenosis at the reimplantation sites of the left common carotid artery and left subclavian artery, with peak systolic velocity acceleration consistent with a hemodynamically significant 70% narrowing. The reconstructed brachiocephalic trunk showed an estimated 80% hemodynamically significant stenosis, with an additional 70% stenosis at the origin of the right subclavian artery. Doppler waveforms revealed marked flow dampening throughout the right carotid axis and evidence of right subclavian steal, with pronounced systolic blunting in the right subclavian artery. Given the absence of neurological symptoms and the preserved perfusion of both upper limbs, these stenoses were managed conservatively with close duplex ultrasound examinations and surveillance, with reintervention to be considered only in the event of progression or the onset of symptoms. Written informed consent for publication for this case report and accompanying images was obtained from the patient.

## Discussion

This case illustrates the potential increased risk in patients with heritable aortopathies and vascular prostheses to severe infectious complications after procedures that are otherwise considered low risk. The progression from lip piercing to prosthetic aortic graft infection and mediastinitis within 3 weeks highlights how the structural and regulatory abnormalities of filamin A amplify the consequences of transient bacteremia. Beyond its cytoskeletal role, filamin A participates in inflammatory signaling, endothelial integrity, and wound repair, and its dysfunction may have contributed to both the accelerated spread of infection and impaired tissue recovery in this patient.^1^^,^^2^^,^^8^

The extensive vascular involvement, from coronary arteries to the popliteal circulation, further underscores the systemic nature of the disorder and its implications for surgical complexity and postoperative fragility. The microbiological findings reinforce the causal link between the piercing and subsequent graft infection. *S aureus*, carried by approximately one-quarter of healthy individuals, entered through the oral piercing site and demonstrated its well-recognized tropism for prosthetic material.^9^ Hematogenous seeding of vascular prostheses from oral or odontogenic sources has been previously reported, particularly in late-onset aortic graft infections involving microorganisms originating from the oral cavity.^10^ In parallel, severe infectious complications including *S aureus* bacteremia and infective endocarditis have been described after orofacial piercings, supporting the plausibility of transient bacteremia after such procedures.^11^ However, prosthetic aortic graft infection secondary to lip piercing has not, to our knowledge, been reported previously.

Baseline oral hygiene and periodontal status were not formally assessed in our patient, which limits inference regarding oral sources beyond the piercing itself. Importantly, the anatomical distance between the lip piercing site and the anterior mediastinum, combined with the circumferential periprosthetic distribution of the fluid collection and the concordant MSSA bacteremia documented at both the referring hospital and our center, strongly favor hematogenous seeding of the prosthetic graft over direct contiguous spread from a cervicofacial source.

This case, therefore, extends existing observations by demonstrating that piercing-related bacteremia may also lead to life-threatening infection of thoracic vascular prostheses, particularly in patients with underlying connective tissue disorders and extensive prosthetic material. The intraoperative identification of a mature biofilm on the explanted Thoraflex and cTAG prostheses explains the limited efficacy of systemic antibiotics despite in vitro susceptibility. Concordant antimicrobial susceptibility profiles between blood cultures and prosthetic isolates confirmed procedure-related bacteremia as the source of infection.^12^ The surgical management reflected the extent of contamination and the unique challenges associated with filaminopathy A. Complete prosthetic explantation was favored over conservative strategies owing to the known tissue fragility in this condition and the associated risk of rupture at smaller diameters.^1^ Reconstruction with cryopreserved allografts was selected for their superior resistance to reinfection in contaminated fields.^13^, ^14^, ^15^

Because cryopreserved allografts can undergo aneurysmal degeneration over time, the supra-aortic trunks were deliberately reimplanted in zone 0 to allow future thoracic endovascular repair if required. This strategy balances radical debridement with preservation of long-term therapeutic options. The use of the Yacoub valve-sparing technique enabled preservation of physiological valve function after removal of all infected tissue. Neurological complications, including recurrent laryngeal and phrenic nerve injury, are consistent with the challenges of reoperative surgery in an inflamed mediastinum. Postoperative pseudoaneurysm formation raised additional considerations regarding tissue healing in filaminopathy A. Although pseudoaneurysms occur in 1% to 5% of aortic surgeries in the general population, their natural history in genetic connective tissue disorders remains poorly defined.^1^ In this case, the lesion regressed and then stabilized, suggesting that close imaging surveillance may be appropriate for small and clinically silent pseudoaneurysms, although optimal thresholds for intervention in heritable aortopathies require further clarification.

Progressive stenoses of the supra-aortic trunks on follow-up imaging, with 70% to 80% narrowing at the anastomotic sites, illustrate the known limitations of cryopreserved allografts and underscore the need for structured long-term surveillance. In this patient, conservative management was chosen given the absence of symptoms, with reintervention to be considered only if stenoses progress or clinical signs develop. Beyond clinical management, this case emphasizes the importance of preventive counseling in patients with heritable connective tissue disorders and vascular prostheses. The absence of prior advice regarding infection risk associated with oral piercings reflects a gap in standard follow-up protocols.^7^ Lifestyle practices that may induce transient bacteremia, such as piercings, tattoos, or invasive dental care, should be discussed explicitly with patients at risk.^5^^,^^9^^,^^16^

Finally, this case underscores the broader organizational demands of treating complex graft infections in rare diseases. Successful management required cross-border access to cryopreserved allografts, coordinated input from cardiac surgery, vascular medicine, and infectious disease specialists, and prolonged postoperative rehabilitation. The resulting health care burden illustrates the importance of preventive strategies in genetically vulnerable populations.

## Conclusions

This case highlights the potential vulnerability of patients with heritable aortopathies, particularly those carrying extensive prosthetic grafts, to severe infectious complications arising from procedures that induce transient bacteremia. Oral piercings, and other lifestyle practices involving skin or mucosal breaches, should be regarded as high risk in individuals with filaminopathy A, and their avoidance should be recommended explicitly. Clinicians managing patients with connective tissue disorders or vascular prostheses must ensure that counseling encompasses not only traditional medical precautions, but also lifestyle procedures such as piercings, tattoos, and invasive dental care. Although radical surgical management with cryopreserved allograft reconstruction successfully eradicated the infection, subsequent complications, including pseudoaneurysm formation and late anastomotic stenosis, reinforce that prevention remains the most effective strategy. This case supports the need for standardized educational protocols and clear prophylactic guidance to decrease the risk of procedure-related infections in patients with genetically mediated aortopathies and vascular prostheses.

## Declaration of generative ai and ai-assisted technologies in the writing process

AI-assisted technologies (Claude 4.5, Anthropic; ChatGPT, OpenAI) were used for manuscript formatting and language editing. All authors reviewed and approved the final version.

## Funding

None.

## Disclosures

None.
